# Giving schools a nudge: can behavioural insights improve recruitment of schools to randomised controlled trials?

**DOI:** 10.1186/s13104-021-05509-8

**Published:** 2021-03-16

**Authors:** Georgina Warner, Fatumo Osman, Serena McDiarmid, Anna Sarkadi

**Affiliations:** 1 Child Health and Parenting (CHAP), Department of Public Health and Caring Sciences, Uppsala University, BMC, Husargatan 3, 751 22 Uppsala, Sweden; 2 School of Education, Health and Social Studies, Dalarna University, Falun, Sweden; 3 Social Development Lab, Department of Psychology, University of Waterloo, Waterloo, ON Canada

**Keywords:** RCT, Recruitment, Schools, Behavioural insights

## Abstract

**Objective:**

It is widely acknowledged that recruitment to randomised controlled trials (RCTs) is challenging, particularly trials that operate across multiple sites. A research area in need of further high-quality evaluation, including RCTs, is school-based mental health interventions for refugee children and adolescents. However, engaging schools with interventions and associated evaluations can be challenging. This paper explores the application of behavioural insights, i.e. evidence-based understanding of how people behave and make decisions, to RCT recruitment at the school level via email communications. A pilot study of applying behavioural insights to mail outs attempting to recruit schools to a RCT of a trauma-focused group intervention for refugee children and adolescents experiencing symptoms of post-traumatic stress is reported. Rates of school involvement between the behavioural insights approach (n = 31) and a standard outreach approach (n = 65) are compared.

**Results:**

Schools were more likely to give a positive response to the mail out designed using the behavioural insights framework than standard outreach. Accounts of recruitment strategies such as this are valuable additions to the literature on RCT methodology given the potential for recruitment issues to affect trial operations.

## Introduction

It is widely acknowledged that recruitment to randomised controlled trials (RCTs) is challenging, particularly trials that operate across multiple sites [2, 7]. A study evaluating factors associated with good and poor recruitment to multicentre trials found that less than one-third recruited their original target within the specified timeframe, and around one-third extended the recruitment period [2]. The most commonly reported strategies to improve recruitment were newsletters and mail outs, but the causal relationship to changes in recruitment was not assessed. A systematic review of methods to improve RCT recruitment reports a number of strategies for recruitment at the individual level, including telephone reminders, opt-out procedures and open designs; however, a clear knowledge gap with regard to effective strategies aimed at “recruiters” was identified [7].

Given the high number of children affected by armed conflict and displacement and the need for more robust evidence to inform the development of best practices guidance, a research area in need of further high-quality evaluation, including RCTs, is school-based mental health interventions for refugee children and adolescents [1]. However, engaging schools with interventions and associated evaluations can be challenging given the active role school personnel are required to take; the processes vary according to the cultural context yet projects often require approval from school leadership, input from language and culture experts, active participation from teachers and school nurses/counselors, and support from administrative personnel [1]. Moreover, for school-based RCTs to be viable, school personnel are sometimes asked to act as “recruiters” to engage the children, adolescents and their families in the trial. We need to know more about effective strategies to engage schools with RCTs, particularly trials evaluating mental health interventions for refugee children.

Initiating new procedures that accompany the decision to participate in a RCT requires behaviour change. Thaler and Sunstein [6] describe how people are influenced by the organisation of the context in which decisions are made, which they term “choice architecture”. The last decade has seen a huge rise in the application of this thinking, with dedicated government departments using inductive approaches to policymaking informed by behavioural insights at an international scale. There are many reported successes [3], which inspires the application of the approach to RCT recruitment.

### EAST framework

The EAST framework focuses on four simple principles to encourage behaviour change: make it Easy, Attractive, Social and Timely (EAST) [4]. The first principle of ‘make it easy’ refers to the simplicity of the process required for behaviour change. Given the strong tendency to go with the ‘default’ option, making the preferred behaviour the default is likely to improve adoption. Alternatively, the effort required to adopt the preferred behaviour can be reduced. An important aspect is the simplicity of communications. Making the message clear often results in a significant increase in response rate [4]. If the desired behaviour change is complex, it should be broken down into simpler, easier actions. The principle of ‘make it attractive’ refers to drawing attention to the communication. Behaviour change is more likely if attention is focused on the behaviour [4]. Attention can be attracted though visual techniques, e.g. use of colour and/or pictures. Using rewards and sanctions tends to maximize effects, with rewards supporting the desired behaviour change and sanctions deterring from taking no action. ‘Make it social’ draws upon collective action. By describing how other people perform the desired behaviour, you can encourage an individual to do the same particularly if the people are within the individual’s network. The final principle of ‘make it timely’ considers when people are most likely to be receptive as well as the timeliness of costs and benefits. Disruptions in habitual routines can be leveraged, and immediate impact has greater effect than delayed impact.

### Objectives

The aims of this paper are to:

1. Investigate the response of preliminary participants when applying the EAST framework to mail outs attempting to recruit schools to a RCT of a trauma-focused group intervention for refugee children and adolescents reporting symptoms of post-traumatic stress.
2. Compare rates of school involvement between the EAST framework approach and a standard outreach approach.

## Main text

### Setting

**Fig. 1:**
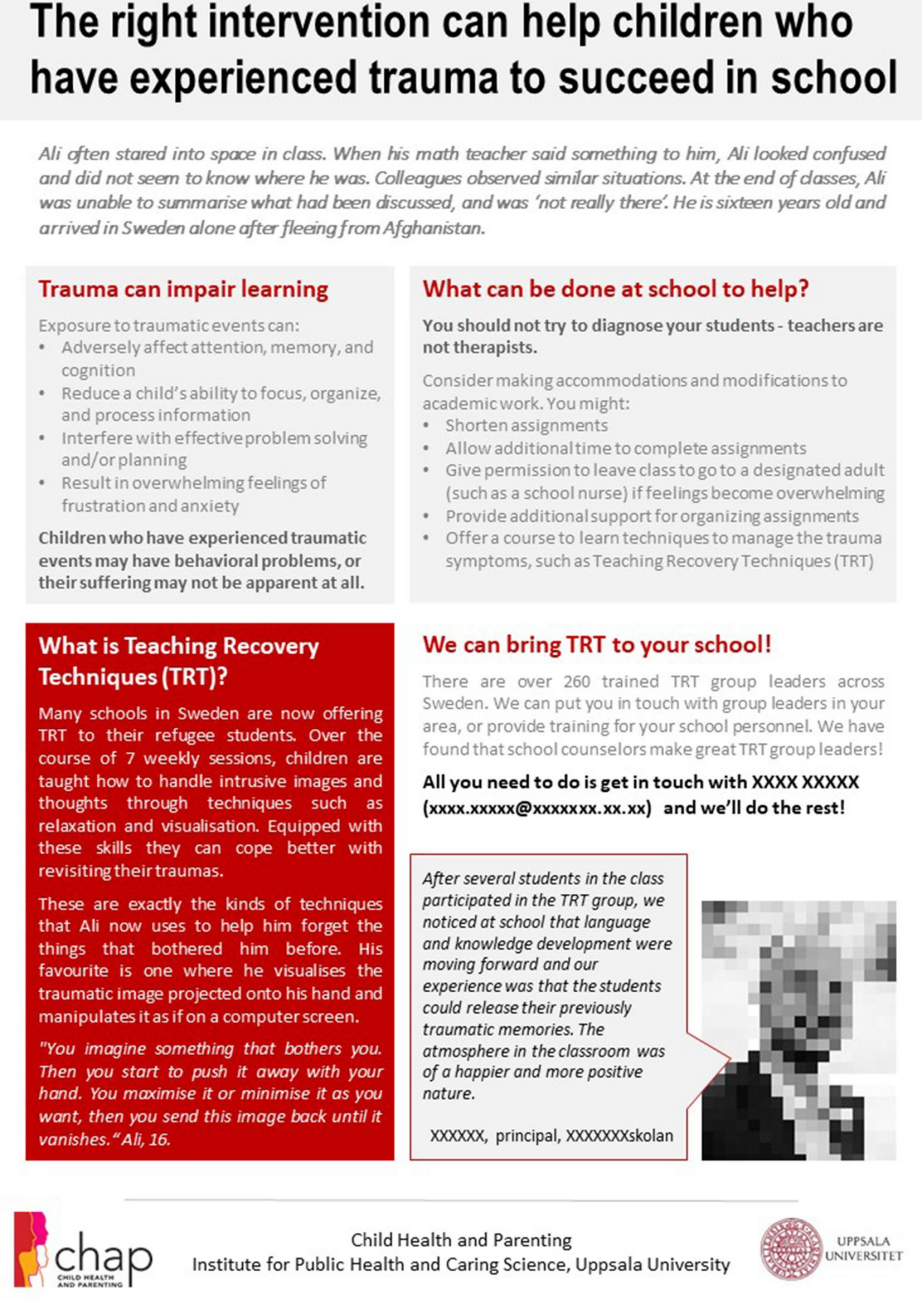
EAST framework school recruitment leaflet (de-identified and translated to English)

Our research group is conducting concurrent trials of a trauma-focused group intervention for refugee children and adolescents reporting symptoms of post-traumatic stress called Teaching Recovery Techniques (TRT). The first is a European Union (EU) wide initiative to improve refugee student wellbeing in schools, which couples TRT with school personnel training on how refugee experiences may impact psychosocial well-being and school functioning. The second is a national Swedish trial of TRT across different settings (including schools, healthcare clinics, and social work departments). Given the substantial likeness between the projects, schools in different areas of Sweden are being targeted. The Swedish school system is tax-financed. In 2020, around 17 per cent of compulsory schools and 34 per cent of upper secondary schools were independent schools with public funding, so-called ‘charter schools’ [5]. Whether the school was independent or run by the municipality did not affect the recruitment approach for the present study. The lead researcher for the EU project first utilized schools that had been involved in her previous research and personal contacts in her home county to secure some trial sites. Beyond this network, she relied on outreach activities such as mail outs and phone calls to get further interest from schools. Only the latter sites, which required outreach activities, were included in the present study. The national Swedish trial team targeted different areas with a mail out, utilising email content and a leaflet designed using the EAST framework (Fig. 1). An initial email was sent followed by two reminder emails, each after two weeks had lapsed, providing the school had not already responded. All communication efforts (school name, contact name, email address, dates of email contacts) and the nature of responses (positive, negative, or no response) were recorded in dedicated files, which were reviewed for this paper. A ‘positive response’ was defined as the school making arrangements for students to be informed and screened. The inclusion criteria for ‘schools contacted’ was at least 30% of students were immigrants and/or introduction classes were offered for newcomer students, taken as indicators of a multiethnic school. Recruitment for the national Swedish trial is ongoing, with a staged approach of targeting new areas. However, only the first round of communications (i.e. the first areas contacted conducted during November 2019 were included in the analysis to allow > 3 months for schools to respond and for trial participation to be underway prior to the more restrictive school policies implemented in response to the COVID-19 pandemic. Once a school agreed to participate, eligible youth and legal guardians (for youth < 15 years old) were provided with information sheets approved by the Uppsala Regional Ethics Board (Refs. 2019-03160; 2018-382) as part of the informed consent process.

### Application of the EAST framework to school mail outs

#### Easy

The communication style was kept simple. Content focused on the need and the intervention. Specific evaluation details were saved for the next communication. A teacher reviewed the content for relevance. Key messages were presented in bold print, including the identified need and the ‘call to action’ of replying to indicate interest in participating. The respondent was able to reply directly to the email address from which the mail out was sent. The mail out was sent to both the school counselor/nurse (who was most likely to be aware of the need for the intervention within the school) and the principal (who was most likely to be the decision maker for introducing new initiatives to the school) in order to facilitate the necessary school-level communications.

#### Attractive

The leaflet was designed with a professional yet bold colour scheme. Content was organized in a visually appealing way, with careful use of subheadings, bullet points and boxed content. An indirect reward approach was adopted though articulating the impact of trauma on learning and a positive testimony from an intervention participant conveying the potential positive impact that could potentially be achieved through the school responding to our call to action.

#### Social

A brief testimony from a principal at a school already delivering the intervention was included, both on the leaflet and in the email, along with a photo of the principal. The purpose of this was to incite peer support in making the decision to be involved. The leaflet also contained a case study describing a refugee boy in class and his apparent attentional difficulties. This was to enable the reader to connect the potential need to the specific context of the classroom, and to children they know personally.

#### Timely

A sense of immediacy was conveyed; the email content clearly stated that the project was already active and that a member of the research team would be in touch as soon as the school responded to the email.

### Results

**Fig. 2:**
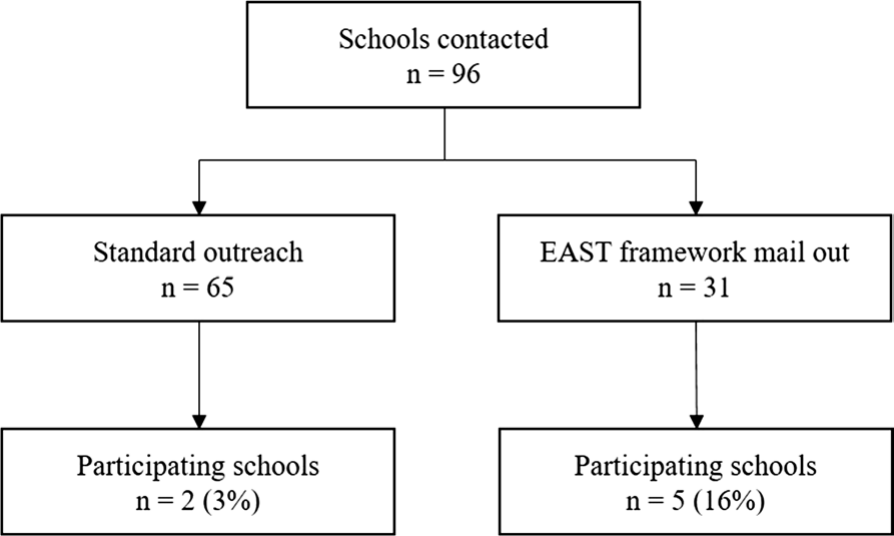
Success rates of outreach approaches

Of the 96 multiethnic schools contacted at the time of analysis, the standard outreach procedure was applied to 65 and the EAST framework mail out procedure was applied to 31. School involvement was achieved for 2 (3%) of the standard outreach schools and for 5 (16%) of the EAST framework mail out schools (Fig. 2). A Fisher’s exact test of independence was performed to examine the relation between the approach made to schools and number that become involved in the trial. The relation between these variables was significant at the 5% level, p = 0.034 (odds ratio = 6.06; 95% CI 1.10, 33.24). Schools were more likely to give a positive response to the mail out designed using the EAST framework than standard outreach.

### Discussion

The alignment of similar RCTs allowed for a natural experiment regarding school recruitment strategies. It appears as though applying the principles of behavioural insights, as detailed in the EAST framework, to school outreach communications can enhance recruitment prospects. Given the perceived success, the team continues to use the strategy. Undeniably, we could see a downturn in its effectiveness as it is utilised more broadly; a situation that is currently complicated by the COVID-19 pandemic. Yet, accounts of recruitment strategies such as this are valuable additions to the literature on RCT methodology given the potential for recruitment issues to affect trial operations. The ethics of applying the EAST framework to research activity need to be recognised. Although researchers may like their project to appear ‘attractive’ it is essential that all aspects relating to participation are made transparent so schools can make an informed choice. The concept of ‘sanctions’, covered in the EAST framework, does not align with the ethical principles of research and schools should *not* be made to feel that choosing to not participate will have adverse effects. Of course, ethical principles should always govern our communications but perhaps a solution to the enduring issue of RCT recruitment is for researchers to become better choice architects?

## Limitations

Recruitment strategies were not randomly allocated to schools, which could have resulted in selection bias. An additional component, i.e. school personnel training, was included in the EU trial, which may have affected participation decisions. One could argue that the additional training within the EU trial constituted a bigger ‘ask’ of the schools; yet, on the other hand, the training was free of charge and so schools received more from the project. Qualitative exploration could have provided greater insight on this, and the recruitment process more broadly, which could have enhanced the generalisability of the findings. The study intended to investigate the recruitment processes within two RCTs conducted in Sweden, hence generalisability of the findings to other international contexts was not an expected attribute and the school system within the local context needs to be considered. Finally, the statistical analysis should be interpreted with caution; the odds ratio was crude and thus could be influenced by other factors and the confidence interval was wide. In addition, the unbalanced group size may have reduced the power of the Fisher’s exact test.

## Data Availability

The data source (i.e. files containing recruitment details) are not publically available due to the personal data they contain.

## Abbreviations

EAST: Easy, Attractive, Social and Timely
EU: European Union
RCT: Randomised controlled trial
